# Investigating the Integration of Nonwoven Carbon Fibers for Mechanical Enhancement in Compression Molded Fiber-Reinforced Polymer Bipolar Plates

**DOI:** 10.3390/polym15193891

**Published:** 2023-09-26

**Authors:** Rainer Frank, Lisa-Maria Wittmann, Tobias Kleffel, Benedikt Roth, Knut Graichen, Dietmar Drummer

**Affiliations:** 1 Institute of Polymer Technology, Friedrich-Alexander-Universität Erlangen-Nürnberg (FAU), Am Weichselgarten 10, 91058 Erlangen, Germany; lisa-maria.lw.wittmann@fau.de (L.-M.W.); tobias.kleffel@fau.de (T.K.); benedikt.roth@fau.de (B.R.); dietmar.drummer@fau.de (D.D.); 2 Chair of Automatic Control, Friedrich-Alexander-Universität Erlangen-Nürnberg (FAU), Cauerstr. 7, 91058 Erlangen, Germany; knut.graichen@fau.de

**Keywords:** compression molding, bipolar plate, fuel cell, fiber reinforcement, carbon fleece

## Abstract

The demand for polymer composite solutions in bipolar plates for polymer electrolyte membrane fuel cells (PEMFCs) has risen due to advantages over metal plates such as longer lifetime, weight reduction, corrosion resistance, flexible manufacturing, freedom of design, and cost-effectiveness. The challenge with polymer composites is achieving both sufficient electrical conductivity and mechanical stability with high filler content. A carbon fiber fleece as reinforcement in a graphite-filled polypropylene (PP) matrix was investigated for use as bipolar plate material with increased mechanical and sufficient conductive properties. Plates with a thickness of 1 mm containing four layers of fleece impregnated in the PP-graphite compound were produced in a compression molding process. Particle and fiber interactions were investigated via microscopy. The plates were characterized with respect to the electrical conductivity and mechanical stability. High electric conductivity was reached for fiber-reinforced and plain PP-graphite compound plates, with increased filler content leading to a higher conductivity. The contact resistance remained largely unaffected by surface etching as no polymeric skin layer formed during compression molding. Fiber-reinforced plates exhibit twice the tensile strength, a significantly higher tensile modulus, and an increased elongation at break, compared to PP filled only with graphite.

## 1. Introduction

The adoption of polymer electrolyte membrane (PEM) fuel cells as a viable alternative to the internal combustion engine in vehicles is hindered by critical challenges related to cost, performance, and durability [1]. One of the major factors that affect durability is the bipolar plate, which comprises up to 75% of the volume and 11–45% of the total cost of a fuel cell stack [2]. The plates play a crucial role in several important functions such as collecting current, separating individual cells, distributing hydrogen and oxygen within cells, removing water from each cell, and cooling the cells.

Consequently, the material used for fuel cell components must possess a combination of high electrical conductivity, adequate mechanical stability, and excellent corrosion resistance. Additionally, it should have low weight and require minimal installation space volume while remaining cost-effective and able to replicate channel structures for the efficient distribution of process gases [3] and cell cooling. In order to satisfy the requirements of the specified profile, ongoing research is being conducted on metallic materials and polymer compound solutions. Metallic bipolar plates possess certain advantages over their composite counterparts, such as high electrical and thermal conductivity, high mechanical strength, and, most importantly, existing pathways for high-volume, high-speed mass production. Yet there are numerous drawbacks such as increased contact resistance and potential corrosion products that may compromise the integrity of the membrane electrode assembly by causing contamination, which is why expensive and complex corrosion coatings are usually applied [1,4].

Polymer-based bipolar plates present a favorable technical and economic substitute due to their notable benefits such as low specific weight, high corrosion resistance [5], durability [6], high functional integration within intricate 3D geometries, and low cost [7]. For this purpose, the used conductive polymer composites require a sufficient electrical conductivity of around 100 S/cm [8], which can be varied over a wide range according to the used filler, such as carbon fiber (CF), graphite (G), or carbon black (CB) and its content [9]. As a matrix material, PP is one of the most widely used polyolefin polymers that can form wet and dry mixtures suitable for compression molding (CM). Due to the electrical insulation of PP, a high filler content is needed. As a result, several studies have been conducted to investigate various filler modifications aimed at achieving high electrical conductivity in bipolar plates [9]. Numerous studies have shown that the level of electrical conductivity is directly proportional to the number of contact points of the individual filler particles that lead to a conductive percolation network and are, thus, strongly dependent on the filler content [10], filler shape, and size [11]. For example, the addition of CB to G fillers produces a synergistic effect, resulting in a significantly higher conductivity in contrast to a singular G filler at the same filler content, by creating interconnected percolation paths between the G particles [12]. For achieving an electrical conductivity of 80 S/cm in polyphenylene sulfide (PPS), a G filler content of around 80 wt.% is required, while the addition of 7 wt.% CB reduces the required G content to 60 wt.% [13]. In PP with a filler content of 80 wt.% G filler in 2 mm thick plates, a conductivity of 15.9 S/cm could be reached [14]. In contrast to particle fillers, continuous CF in the form of fabric, mats, or fleece forms an intrinsic percolation network and, thus, leads to high in-plane electrical conductivity [15]. 

Besides the effect on electrical properties, high filler contents also result in a change in the mechanical property profile. This is of particular interest for the handling of bipolar plates during stack assembly. Recent studies on the influence of the filler content of G-filled PP sheets on their mechanical properties show an increase in the modulus of elasticity from approx. 1600 MPa with unfilled PP to up to 5800 MPa with 60 wt.% G content, whereas the elongation at break of the composite already drops from 420% to 4% with the addition of 10 wt.% G [14]. This brittle material behavior has a particularly negative effect on the mechanical behavior under bending or impact bending stress and must therefore be regarded as critical for handling and stack assembly.

To enhance mechanical properties, continuous fibers can be integrated. CM holds the potential of preserving the integrity of continuous fibers, as the process induces low shear stress and thereby minimizes their destruction during manufacturing [16]. Additionally, due to lower shear rates compared to injection molding and therefore the absence of particle segregation in CM, a polymer-rich skin layer is less pronounced. Hence, the necessity for post-processing methods like surface etching, commonly employed to uncover particles and minimize surface resistance, is diminished [17]. Further advantages over injection molding include low tooling costs due to the simplicity of the process [18], reduced wastage, and reduced post-processing requirements resulting from the absence of a gating system [19]. However, CM does have limitations. In relation to the bipolar plate, this is specifically the limited production speed while the limitation to flat or moderately curved parts without undercuts is not critical [20]. The combination of highly filled polymers with fiber fleece has not been investigated in relation to conductivity and mechanical stability.

The aim of the present study is to investigate the influence of an endless fiber reinforcement on the electrical and mechanical properties of polymer-based bipolar plates using a CM process. For the investigation, a highly filled PP with G in combination with a carbon fleece as an endless fiber reinforcement was used. The electrical conductivity of the produced fleece composites was determined through plane, in comparison to plates made of only highly filled PP with G. In addition, the influence of a polymeric skin layer was analyzed by SEM images and measurements of the electrical conductivity before and after etching the surfaces via oxygen plasma. The particle orientation and fleece impregnation were studied with bright field microscopy. Furthermore, the mechanical properties of the resulting composites were tested to evaluate the reinforcement influence of the carbon fleece.

## 2. Materials, Specimens, and Methods

### 2.1. Materials

Compound PP BJ100HP (Borealis AG, Vienna, Austria) and flake-shaped graphite particles of the type GraphCOND (Georg H. LUH GmbH, Walluf, Germany) with a percentile value of D90: 50–70 µm were mixed with 70 and 80 wt.%-proportions by a twin-screw extruder of the type ZSE HP 27 (Leistritz Group, Nuremberg, Germany). The nozzle temperature was set to 270 °C to reduce viscosity. At a screw speed of 150 rpm, a dosing speed of up to 1.0 kg PP and 4.0 kg of Graphite was reached. The compound was then cryogenically ground with a Thermomix type TM6 (Vorwerk SE & Co., KG, Wuppertal, Germany) at 10,000 rpm for 2 min, after adding liquid nitrogen inside the mixing bowl.

For the CF fleece, a low areal weight of 20 g/m^2^ was selected to minimize any potential filler segregation during the impregnation process. The fleece from R&G GmbH, Waldenbuch, Germany, has a thickness of 0.24 mm at 10 kPa, and the single high-tenacity (HT) fibers have an average nominal thickness of 7 μm. The integrity of the fleece is provided by a polyvinyl alcohol (PVOH) binder. 

### 2.2. Specimens

For the investigation of the electrical conductivity and mechanical characterization, specimens were prepared in the following configurations: pure polypropylene (PP), PP with fleece (PP + CF), PP graphite compound (PP + G), and PP graphite compound with fleece (PP + G + CF). The graphite concentration in the compound was 70 and 80 wt.% for the different configurations, respectively.

**Figure 1:**
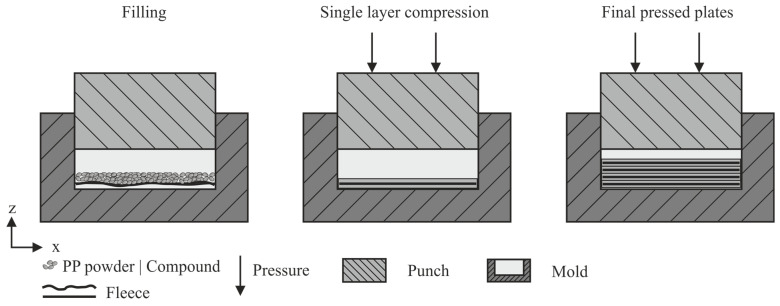
Schematic pressing process for 1 mm plates consisting. The partial steps, consisting of filling the mold, compressing into single layers, and combining four single layers into one plate.

A total of 60 × 60 × 1 mm^3^ plates were produced via CM with a hydraulic hot press A DDP 850 (ATM Deutschland Maschinen & Werkzeuge Vertriebs GmbH, Blieskastel, Germany). The stamp and the mold are heated electrically. After pressing, the die can be dislocated manually and the formed plates are cooled to room temperature with a water-cooled stamp under pressure. The pressing process took place under defined temperature and pressure conditions chosen to assure sufficient material flow and impregnation, which are shown in Table 1. The fiber-reinforced samples were manufactured in multiple stages, which are displayed in Figure 1. A layer of nonwoven fabric with a size of 60 × 60 mm^2^ and an areal weight of 20 g/m^2^ was placed into the mold, covered with 2.0 g of 80 wt.%, 2.3 g of 70 wt.% ground compound or 0.8 g PP, respectively, and then compressed into a single layer of 0.25 mm. Four layers each were subsequently pressed together to form a plate with a thickness of 1 mm. The samples made from pure graphite compound were prepared by the same procedure, only without the fleece. The systematic process assured a homogeneous plate thickness and contributed to a good compound consolidation.

### 2.3. Thermogravimetric Analysis

The filler content in the compounding process was assessed using a thermogravimetric analysis conducted with a TGA Q5000 instrument (TA Instruments, New Castle, DE, USA) under nitrogen atmosphere. The filler content was determined after the thermal decomposition of the polymer at a temperature of 500 °C. To ensure that no decomposition of the graphite occurred or carbonaceous residues of the PP distorted the result, the pure graphite powder and the pure PP were measured accordingly.

### 2.4. Microscopic Analysis

A microscope Axio Imager.M2 (Carl Zeiss AG, Oberkochen, Germany) was used to evaluate the graphite and fleece interaction in the cross-section of the plate center. Therefore, samples were embedded in epoxy resin, ground, and polished by hand. For detecting the influence of the surface layer on the electrical conductivity, the sample surface of the unetched and etched specimens was observed via scanning electron microscope (SEM) of type Gemini Ultra-Plus (Carl Zeiss AG, Oberkochen, Germany).

### 2.5. Electrical Conductivity

**Figure 2:**
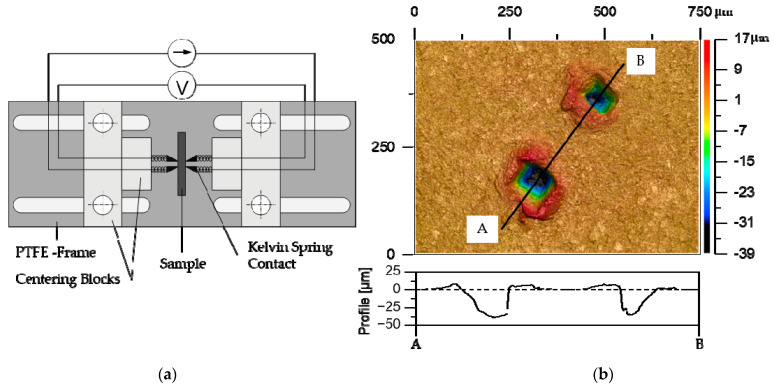
(**a**) Schematic setup of the conductivity measurement. (**b**) Measuring point distance based on optical images.

The through-plane electrical conductivity was measured on plate cutouts measuring 50 × 50 mm^2^ using a four-point measurement setup with Kelvin spring contacts BA10 (FIXTEST Prüfmittelbau GmbH, Engen, Germany) with square pyramidal tips of 1 mm^2^ base size and an electrode distance of 0.2 mm. A Keithley 6220 (Keithley Instruments, Cleveland, OH, USA) was used as precision current source, and a Keithley 2182A (Keithley Instruments, Cleveland, OH, USA) served as nanovoltmeter. To ensure consistent pressure and parallel alignment of the Kelvin contacts, a centering block made from Polytetrafluorethylen (PTFE) was employed to hold the measuring tips in place, as shown in Figure 2a. The blocks can be moved along the elongated holes in the frame, compressing the spring of the Kelvin contacts until the block front plane rests on the sample plate, holding it into place, and assuring a plane-parallel alignment and a reproducible electrode pressure. 

In order to determine the specific conductivity *σ,* the reciprocal value of the specific resistivity *ρ* was calculated using Equation (1). The actual cross-sectional area (A) of the current passage in the plate was measured based on the edge length of the electrode imprint, using a laser scanning microscope type VK-X1000 (Keyence Corporation, Osaka, Japan) with a 20-fold magnification and the laser measuring, as shown in Figure 2b. The plate thickness *d* at the contacting points was measured with an outside micrometer.

(1) $$\sigma =\frac{1}{\rho }=\frac{1}{R}*\frac{d}{A}$$

Prior to measuring, the plates were cleaned by hand using 2-propanol (IPA). The measurements were repeated after O_2_ plasma etching of the surface with the device Tepla 440 (Technics Plasma GmbH, Kirchheim, Germany) 5 times for 5 min at 500 W. The treatment was applied to remove isolating, polymer-rich surface layers and thereby detect any potential contact resistance.

### 2.6. Mechanical Properties

The tensile strength, Young’s modulus, and elongation at break were characterized using tensile tests on a tensile testing machine of the type MicroTester 5948 (Instron GmbH, Darmstadt, Germany) according to DIN EN ISO 527-1, -2. The test speed for determining Young’s modulus was 0.2 mm/min, and the test speed to break was 10 mm/min. The samples were cut from the center of the plate, with dimensions of 60 mm × 10 mm × 1 mm. Since the specimens have a constant width, the risk of failure at the clamping points is increased. A reinforcement in the form of aluminum strips was applied to the ends of the specimens. Commercially available superglue of type Pattex Sekundenkleber Plastik (Henkel AG & Co. KGaA, Duesseldorf, Germany) was used as the adhesive.

## 3. Results and Discussion

### 3.1. Filling Content

**Figure 3:**
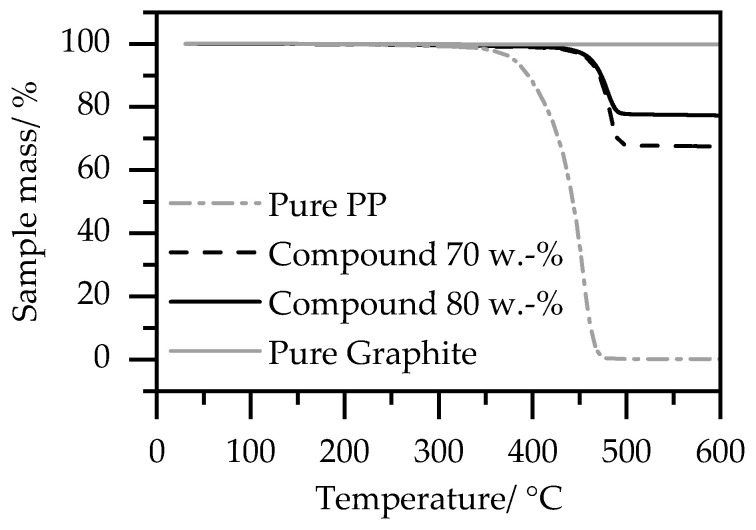
Thermogravimetric analysis of the pure PP and pure graphite, and of the compound with 70 wt.% and 80 wt.%.

Figure 3 shows the results of the thermogravimetric analysis of the pure PP and pure graphite in comparison to the compound with a maximum calculated filler content of 70 wt.% and 80 wt.%.

While there is no residue for the pure PP above 500 °C, the graphite does not decompose in a temperature range until 600 °C.

The results show that with the chosen parameter set, the desired weight fractions of graphite were achieved with only minor deviations. For a desired filler content of 80 wt.%, 76 wt.% is in fact obtained, and with 70 wt.%, 66 wt.% is achieved. 

### 3.2. Optical Analysis

**Figure 4:**
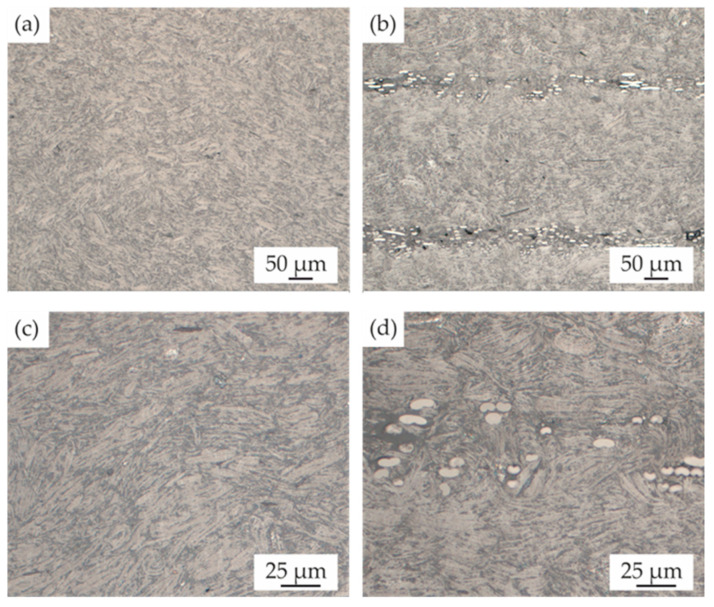
Results of the hot-pressed plates, PP filled with 80 wt.% graphite (**a**,**c**) and PP filled with 80 wt.% graphite and carbon fleece (**b**,**d**).

Bright-field microscopy images were used to evaluate the graphite and fleece interaction in the cross-section. Figure 4a,b show overview images, and c and d show enlarged details. Since the basic interaction between compound and fleece is independent of the filler content, only exemplary images with a filler content of 80 wt.% are shown. 

No major displacement of the fleece during the impregnation process, which could compromise the resulting mechanical properties, can be detected. The graphite orientation can be observed clearly but does not seem to follow a distinct pattern, both in the case of the pure graphite plate and in the graphite fleece combination. Yet a clear difference in structure can be seen. Upon contact with the fleece, a separation of the matrix and graphite is noticeable. A filler-deficient zone exists between the carbon fibers. The nonwoven fabric appears to act as a sieve, retaining the graphite during consolidation.

**Figure 5:**
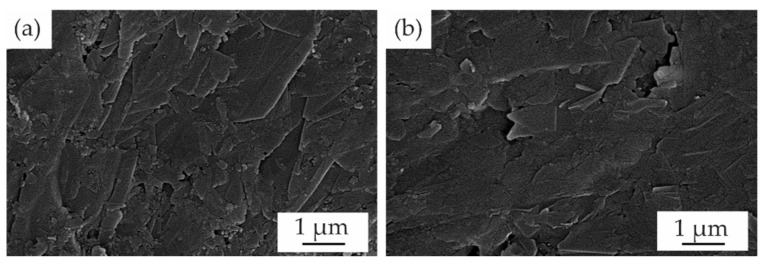
SEM images of the surface structure of plates with the 80 wt.% graphite compound (**a**) before and (**b**) after etching. No segregated polymer layer can be observed or removed.

The surface before and after plasma etching is shown for the 80 wt.% graphite compound in Figure 5. Single graphite particles are prominent even for the untreated surface, while a marginal additional exposure of the particles on the surface occurs during etching. The particles align mainly with the surface of the mold; the orientation remains unchanged during the treatment.

### 3.3. Electrical Conductivity

**Figure 6:**
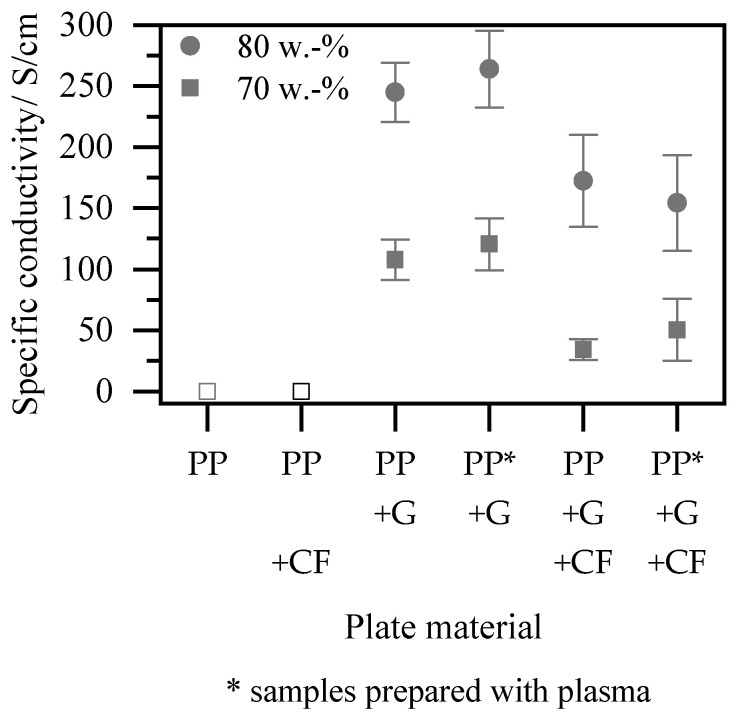
Results of the specific conductivity for different plate configurations.

Figure 6 shows the results of the electrical measurements on plate specimens with varying graphite content, with and without fleece. Pure PP shows a complete insulating behavior. The addition of fleece does not result in a measurable specific conductivity through plane, as the carbon fibers do not penetrate the polymer surface and cannot be contacted. By adding 70 wt.% graphite, sufficient particle percolation is achieved, resulting in a high specific conductivity of over 100 S/cm. Increasing the graphite content to 80 wt.% significantly enhances the specific conductivity to almost 250 S/cm. The addition of fleece to the 80 wt.% compound leads to a reduction in conductivity of 30%, yet the required 100 S/cm for use in PEM [3] can still be exceeded. For the 70 wt.% graphite compound, the incorporation of fleece leads to a greater reduction of almost 70%. This can be attributed to the observed matrix filler separation in Section 3.2, where less consolidation seems to be reached for the 70 wt.% graphite. To avoid this effect in the future, a thorough investigation of particle interaction with carbon fibers, considering particle size and distribution as well as fiber arrangement, is necessary. 

Etching the surface of the samples, on the other hand, has little effect on the specific conductivity, regardless of the configuration, as shown in Figure 6. In agreement with the observations described in Section 3.2, this suggests that, in contrast to the injection compression molded samples investigated [17], there is hardly any protective polymer surface layer present. Eliminating the necessity of a post-processing step would offer significant advantages for an industrial process.

### 3.4. Mechanical Properties

**Figure 7:**
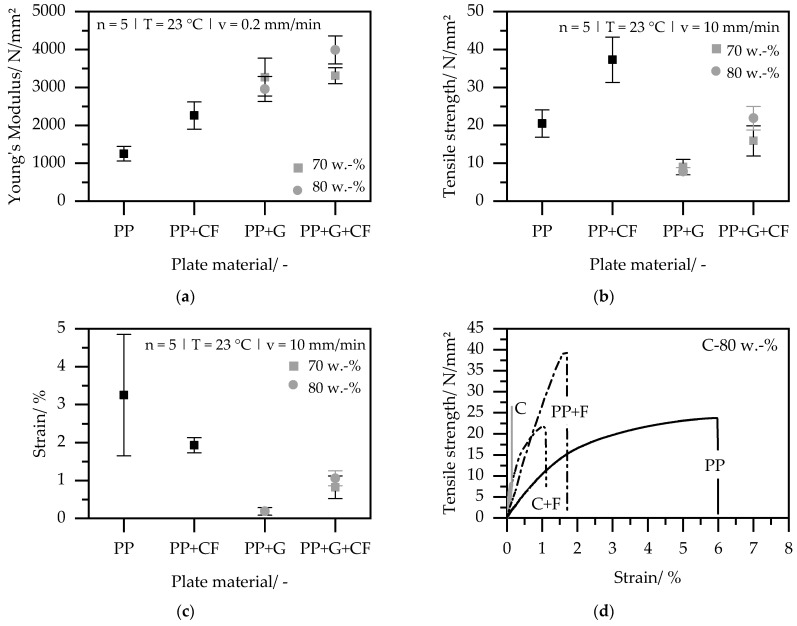
Results of the mechanical properties: (**a**) Young’s modulus; (**b**) tensile strength; (**c**) Elongation at break; (**d**) Comparison of the Stress–Strain development of specimen made from PP, PP + G, PP + CF, and PP + G + CF.

The results of the mechanical characterization are plotted in Figure 7. In Figure 7a, Young’s modulus is shown; in Figure 7b, the tensile strength; and in Figure 7c, the maximum achievable strain. In addition, Figure 7d exemplarily shows the stress–strain development curves for 80 wt.%.

Young’s modulus is significantly influenced by the presence of the graphite compound and the carbon fleece. Young’s modulus for pure PP plates is around 1250 ± 180 N/mm^2^. The test specimens made of PP-compound with 70 wt.% and 80 wt.% graphite achieve 3270 ± 500 N/mm^2^ and 2960 ± 330 N/mm^2^, respectively, and thus have a 2.4–2.6 times higher stiffness compared to the reference material. If the pure PP is compressed with carbon fleece, a Young’s modulus of 2260 ± 360 N/mm^2^ is reached, which corresponds to an enhancement by a factor of 1.8. With a combination of PP-compound with 80 wt.% graphite and carbon fleece, Young’s modulus is 3990 ± 370 N/mm^2^ and, thus, three times higher than that of pure PP. The reinforcing effect of the particles and fleece complement each other, as the particles do not appear to hinder the integrity of the fibers.

Regarding the tensile strength, the presence of the fleece has a clearly positive effect. While the pure PP achieves a low tensile strength of less than 20 ± 3.6 N/mm^2^ (25 MPa according to the datasheet), the combination of pure PP and fleece approximately doubles the strength to 37 ± 6 N/mm^2^. The inclusion of graphite reduces the tensile strength to 9 ± 2 N/mm^2^ for 70 wt.% and 8 ± 1 N/mm^2^ for 80 wt.%, respectively. With the combination of PP-compound and fleece, values at the level of the pure PP are achieved and, thus, twice-higher tensile strengths are obtained compared to PP-compound. The positive influence of the fleece is also apparent in the elongation at break. PP-compound breaks brittle immediately. The combination of PP-compound and fleece, however, achieves slightly higher elongations at break of around 1%. Poor adhesion mechanisms between the fillers and the matrix act as notches and starting points for cracks, which deteriorate the tensile strength, elongation at break, and flexibility of the composite. By supporting the compound with a multidirectional reinforced carbon fleece, applied loads can be transferred from the polymer matrix to a part of the fibers via shear stresses, resulting in an increase in stiffness and tensile strength compared to unfilled polymer systems.

## 4. Conclusions

This study explores the potential of fabricating highly conductive, thin-walled bipolar plates using compression molding (CM) of fiber-reinforced polymer composites. The main results are listed in the following.

1. High electrical conductivity through the incorporation of graphite, up to 250 S/cm at 80 wt.% filler content, was achieved. While the addition of fleece introduces some reduction in conductivity, the target conductivity thresholds of 100 S/cm for utilization as PEM material can still be surpassed, highlighting the potential practical viability of the composite material.
2. The small difference in measured conductivity before and after surface etching indicates that the process yields minimal polymer surface layer formation, underscoring the inherent potential of the CM process.
3. Significant enhancements are observed in the mechanical properties through the incorporation of nonwoven carbon fibers. When the compound is combined with carbon fleece, Young’s modulus becomes three times higher than that of pure polypropylene (PP), reaching up to 4000 N/mm^2^. Although the tensile strength is greatly reduced by a high graphite content, the addition of fleece effectively restores the strength to levels comparable to pure PP of 20 N/mm^2^. The same applies to the elongation at break, which is significantly reduced by high graphite content and can be improved again by the fleece.

Furthermore, upcoming studies should investigate the potential of rearranging the fabric or employing different carbon fiber materials to establish conductive pathways, thereby enhancing conductivity and mechanical stability. Additionally, the potential of continuous fiber reinforcement for enhancing long-term behavior under dynamic mechanical and thermal loading needs to be explored. To address the constraints associated with the discontinuous pressing process, ongoing research is focused on exploring alternative continuous pressing methods. These efforts aim to facilitate the high-volume manufacturing of thin-walled composite polymer bipolar plates at low costs and short cycle times.

## Figures and Tables

**Table 1:** Main process parameters of the production of the plate specimens for the determination of the electrical conductivity and mechanical stability.

Parameter [Unit]	PP and PP with Carbon Fleece (PP + CF)	Graphite-Filled PP (PP + G)	Graphite-Filled PP with Carbon Fleece (PP +G + CF)
Mold temperature (heating) T_m_ (°C)	200	250	250
Heating time t_h_ (s)	300		
Mold temperature (cooling) T_m_ (°C)	50		
Cooling time t_c_ (s)	300		
Compression pressure F_c_ (bar)	250		
Demolding temperature T_d_ (°C)	50		

## Data Availability

The data presented in this study are available upon request from the corresponding author.
